# miRNA profiling during antigen-dependent T cell activation: A role for miR-132-3p

**DOI:** 10.1038/s41598-017-03689-7

**Published:** 2017-06-14

**Authors:** Cristina Gutiérrez-Vázquez, Ana Rodríguez-Galán, Marcos Fernández-Alfara, María Mittelbrunn, Fátima Sánchez-Cabo, Dannys Jorge Martínez-Herrera, Marta Ramírez-Huesca, Alberto Pascual-Montano, Francisco Sánchez-Madrid

**Affiliations:** 1 Instituto de Investigación Sanitaria Princesa, Hospital Universitario de la Princesa, Universidad Autónoma de Madrid, Madrid, Spain; 2 Fundación Centro Nacional de Investigaciones Cardiovasculares Carlos III (CNIC), Madrid, Spain; 3 Centro Nacional de Biotecnología-CSIC, Madrid, Spain; 4 CIBER Cardiovascular, Madrid, Spain

## Abstract

microRNAs (miRNAs) are tightly regulated during T lymphocyte activation to enable the establishment of precise immune responses. Here, we analyzed the changes of the miRNA profiles of T cells in response to activation by cognate interaction with dendritic cells. We also studied mRNA targets common to miRNAs regulated in T cell activation. *pik3r1* gene, which encodes the regulatory subunits of PI3K p50, p55 and p85, was identified as target of miRNAs upregulated after T cell activation. Using 3′UTR luciferase reporter-based and biochemical assays, we showed the inhibitory relationship between miR-132-3p upregulation and expression of the *pik3r1* gene. Our results indicate that specific miRNAs whose expression is modulated during T cell activation might regulate PI3K signaling in T cells.

## Introduction

T cells display specific miRNA profiles compared to other cell types of the immune system. These profiles show specific changes in response to activation by CD3 and CD28 antibody stimulation and T helper (Th) cell *in vitro* polarization^1–3^. Studies using mice deficient for genes involved in the miRNA biogenesis pathway, e.g. Dicer and Drosha, established the central role of miRNAs in the regulation of development and homeostasis of the immune system, and specifically Th cell differentiation^4–6^.

miR-132-3p has been mainly described in the nervous system, with only a few recent emerging examples in the immune system, e.g. regulation of hematopoietic stem cell function^7^. miR-132-3p facilitates viral infection both in innate immune cells^8^ and CD4 T cells^9^. Moreover, the miR-132/212 cluster has been described in the interphase between nervous and immune systems since it is related to resistance to experimental autoimmune encephalomyelitis (EAE). Hence, miR-132/212 cluster induces a cholinergic anti-inflammatory effect on EAE by targeting acetylcholinesterase upon aryl hydrocarbon receptor activation by its exogenous ligand TCDD^10–12^. Recently, the miR-132/212 cluster has been involved in B cell development when it is induced in response to B cell receptor and targets Sox4^13^. However none of these works studied the role of miR-132-3p in CD4 T cell activation.

Here we used a miRNA microarray approach to study the miRNA profile of T cells after their encounter with professional antigen-presenting cells (APC) bearing specific antigen. We then studied *in silico* the mRNAs predicted to be targeted by the combination of the miRNAs upregulated after T cell activation. We observed that the mRNA and protein levels of the *pik3r1* gene displayed a negative correlation with specific miRNAs upregulated during T cell activation. Finally we established the direct inhibition of *pik3r1* by miR-132-3p, one of the miRNAs upregulated after T cell activation.

## Results and Discussion

### miRNA profile of CD4+ T cells after cognate interactions with antigen-loaded dendritic cells

**Figure 1:**
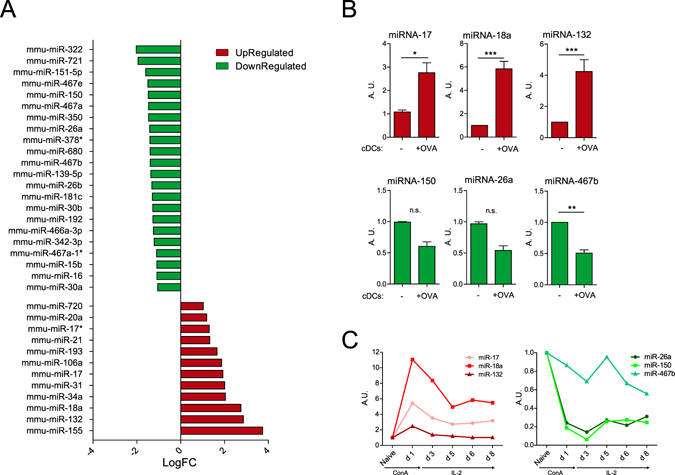
microRNA profile of CD4 T cells activated by cognate interaction with cDCs. CD4 T cells from OT-II mice were cocultured with cDCs in the presence or absence of OVA peptide for 18 h and their miRNA analyzed by miRNA microarray. **(A)** Comparison of miRNAs expression on T cells after stimulation by cDCs loaded or not with OVA peptide. **(B)** Selected miR-17-5p, miR-18a-5p, miR-132-3p, miR-26a-5p, miR-150-5p and miR-467b-5p miRNAs were validated by RT-qPCR. miRNAs that were detected by microarrays to be upregulated or downregulated are depicted in red and green respectively. (n = 8) **(C)** miRNA levels were assessed by RT-qPCR in freshly isolated mouse naive CD4 T cells in different time points of activation with ConA followed by IL-2. (Plots are representative of two independent experiments). RNU1A1 and RNU5G were used as endogenous controls and data are presented in arbitrary units (A.U.). ***P < 0.001; **P < 0.05; ns, non-significant.

To analyze the miRNA profile of CD4 T cells after their encounter with an APC, we co-cultured freshly isolated CD4 T cells from OT-II transgenic mice with *in vitro* derived conventional dendritic cells (cDCs) in the presence or absence of chicken ovalbumin (OVA) 323–339 peptide. CD4 T cells from OT-II mice express a T cell receptor that is specific for OVA peptide in the context of I-A b. After 18 h of co-culture, CD69 and CD25 activation markers were upregulated in T cells after co-culture with cDCs in the presence of OVA but not in its absence (Supplementary Figure S1A). The effect was similar to the one observed using antigen-independent stimulation with antibodies against CD3 and CD28 (Supplementary Figure 1B). CD4+ T cells were subsequently sorted from the coculture by flow cytometry and their miRNA profile analyzed using Agilent microarrays. We found 34 miRNAs differentially expressed in T cells activated by cDCs-OVA compared to those co-cultured in the absence of OVA (Fig. 1A). Our microarray data agree with previous studies on miRNA profile changes on T cells after activation with CD3 and CD28 antibodies assessed by different techniques, from Northern blot to microarrays^3, 14–16^. Our study adds information regarding the modulation of the T cell miRNA profile using a more physiological trigger, i.e. antigen-loaded professional APC. This is likely to better represent an *in vivo* scenario of T cell activation.

We next performed spot-check validation of some of the observed miRNA found in the microarray data by RT-qPCR. We chose miRNAs previously known to be implicated in T cell activation like miR-17-5p, miR-18a-5p and miR-150-5p as well as others not previously described to be related to this process (miR-132-3p, miR-467b-5p and miR-26a-5p). miR-17-5p, miR-18a-5p and miR-132-3p were significantly upregulated in T cells upon contact with OVA-loaded cDC (Fig. 1B). They were also upregulated, albeit to a lower extent, when T cells were stimulated with OVA-loaded plasmacytoid DCs (pDC) (Supplementary Figure S2). miR-467b-5p was significantly downregulated in T cells upon contact with OVA-loaded cDC (Fig. 1B). miR-26a-5p and miR-150-5p also exhibited lower levels when pulsed with OVA-loaded cDCs and pDCs (Fig. 1B and Supplementary Figure S2).

The expression levels of the identified miRNAs were also assessed in CD4 T cells treated with the polyclonal activator concanavalin-A (ConA) followed by expansion with recombinant interleukin 2 (IL-2). A clear upregulation of miR-17-5p, miR-18a-5p and miR-132-3p was detected. The three miRNAs were maximally expressed at 24 h post stimulation. Interestingly, the levels of miR-132-3p were back to the baseline after approximately 96 h. Conversely, the levels of miR-17-5p and miR-18a-5p remained elevated after eight days (Fig. 1C). Similar behaviors were observed in those miRNA that became downregulated in response to ConA. For example, miR-26a and miR-150 decreased sharply their levels only 24 h post stimulation, reaching a lowest value after 72 h and remaining low up to eight days. Conversely, miR-467b-5p displayed a minor decrease after 72 h, and its levels fluctuated afterwards (Fig. 1C). Overall, these data indicate that there is a specific profile of miRNAs regulated after T cell activation. Also, we found similarities of the miRNA profiles changes triggered by antigen-loaded cDC and the mitogen ConA.

### PIK3R1 is a target of the miRNAs upregulated during T cell activation

As a first approach to identify the possible common mRNA targets of the miRNAs that were regulated after T cell activation, we used a customized program which combines several prediction algorithms as well as databases for experimentally validated targets making its prediction more reliable^17^. Interestingly, several target genes of the miRNAs upregulated after T cell activation, were related to the establishment of the immune response (Supplementary Figure S3). We focused our attention on those mRNA targets that have the higher number of predicted miRNAs modulating them, particularly those interactions that have not been experimentally validated yet. Table 1 shows some of these genes and the combined prediction score of our prediction tool.

**Table 1:** Targets of upregulated miRNAs in CD4 T cell activation.

Gen	Number of miRNAs	Combined Prediction SCORE
Tnrc6b	12	0.80847
Eif4g2	11	0.73535
Tnrc6a	11	0.68594
Sox5	11	0.66711
**Pik3r1**	**11**	**0.65147**
Tbc1d2b	11	0.59698
Tbxas1	11	0.57699
Syncrip	11	0.56623
Gsk3b	11	0.56402
Neo1	11	0.56114
Zfp664	11	0.54515
Rdx	11	0.53015

Prediction of the targets of the miRNAs upregulated in CD4 T cells from OT-II mice after stimulation with cDCs loaded with OVA peptide. Prediction was performed with a combinatorial method of different available prediction tools. Higher combined prediction score denotes more confidence in the prediction.

We focused in the *pik3r1* (Phosphoinositide-3-Kinase, Regulatory Subunit 1 Alpha) gene among those predicted targets of the miRNAs upregulated after T cell activation since it is implicated in important signaling pathways of this process. Interestingly, *pik3r1* was predicted to be inhibited by 11 out of the 12 miRNAs upregulated in T cells after cognate interaction with cDCs (Table 1). The specific list of these miRNAs and their logFoldChange of cDC- vs cDC-OVA stimulated CD4 T cells is presented in Table 2.

**Table 2:** miRNAs upregulated in CD4 T cell activation.

miRNA	logFC	adjpv
mmu-miR-155-5p	3,746	0,003
mmu-miR-132-3p	2,865	0,031
mmu-miR-18a-5p	2,752	0,025
mmu-miR-34a-5p	2,041	0,012
mmu-miR-31-5p	2,012	0,042
mmu-miR-17-5p	1,928	0,008
mmu-miR-106a-5p	1,867	0,018
mmu-miR-193a-3p	1,661	0,018
mmu-miR-21a-5p	1,330	0,042
mmu-miR-17-3p	1,307	0,039
mmu-miR-20a-5p	1,190	0,031
mmu-miR-146a-5p	0,936	0,048

A total of 11 out of the 12 miRNAs upregulated after CD4 T cell stimulation by cDC-OVA are predicted to be targeting pik3r1. The only miRNA not predicted to target is presented in gray.

*Pik3r1* encodes for the proteins p50α, p55α and p85α, which are the regulatory subunits of Class IA phosphatidylinositol 3-kinases (PI3K). PI3K signaling pathway is one of the signaling pathways that arise from TCR and co-receptors engagement during T cell activation. The main role of the regulatory subunits of I_A_ PI3K is to bind and stabilize the catalytic subunit p110, inhibiting its activity in basal conditions^18, 19^. They also recruit the PI3K complex to phosphotyrosine residues in receptors and adaptor molecules, which relieve the inhibitory contact with the catalytic subunits and will bring them in contact with their lipid substrates in the membrane^20^.

**Figure 2:**
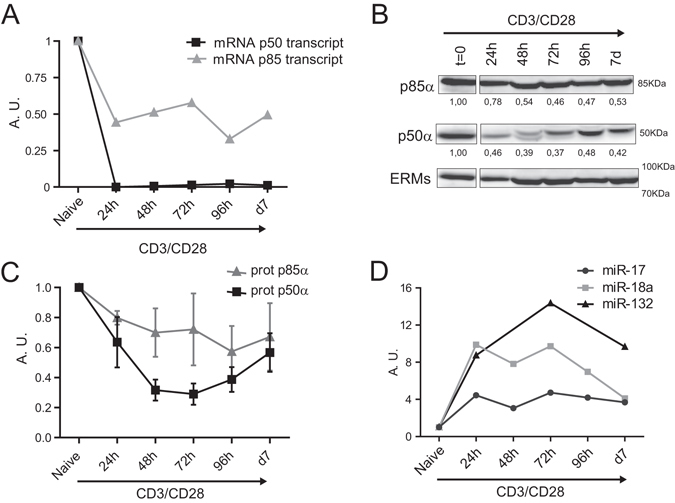
pik3r1 is downregulated during T cell activation. **(A)** mRNA relative levels of the two main transcripts of *pik3r1* were measured by qPCR. Levels were normalized to Yhwaz and β-actin housekeeping genes (n = 2). **(B)** Western blot analysis of p85α and p50α protein content in CD4 T cells after activation with anti-CD3 plus anti-CD28. Representative Immunoblots (n = 3); protein bands were cropped from the same gel. ERMs were included as a loading control. **(C)** Protein levels of p85α and p50α in (B) normalized to ERMs. **(D)** miRNA levels in CD4 T cells after activation with anti-CD3 plus anti-CD28. Levels are normalized to RNU1A1 and RNU5G and presented in arbitrary units (n = 2).

The expression of the corresponding proteins and mRNA of *pik3r1* gene were studied during T cell activation. p85 and p50 proteins, and the mRNA levels of the two corresponding alternative transcripts, decreased over time in CD4 T cells stimulated with anti CD3 and anti CD28 for 7 d (Fig. 2A,B and C). On the other hand, miR-17-5p, miR-18a-5p and miR-132-3p, which are predicted to target *pik3r1*, were inversely regulated (Fig. 2D). These data indicate that the regulator of PI3K, *pik3r1*, might be directly modulated by miRNAs induced during T cell activation.

### miR-132-3p targets *pik3r1*

**Figure 3:**
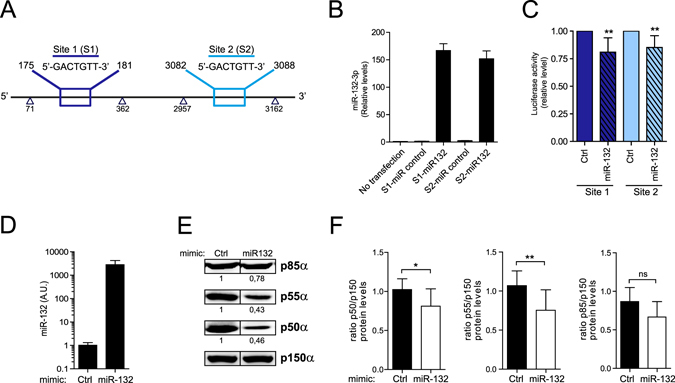
miR-132-3p targets pik3r1. **(A)** 3′UTR of *pik3r1* cloning strategy. Fragments from 71 to 362 bp and from 2957 to 3162 bp containing the two binding sites predicted for miR-132-3p were cloned into the psiCheck2 vector. **(B)** miR-132-3p levels in HEK cells after transfection. Levels were normalized to RNU1A1 and RNU5G. **(C)** HEK cells were transfected with indicated plasmids (Control empty vector or miR-132 expressing vector), GFP+ cells sorted and Renilla and Firefly luciferase signal measured. Data are presented in Renilla Luciferase signal relative to Firefly (n = 5). T-test **P < 0.05. **(D)** miR-132-3p expression in Jurkat cells 48 h post transfection with either negative Control-Dy547 or mmu-miR-132-3p miRNA Mimics. **(E)** Western blot analysis of p85α, p55α and p50α isoforms in Jurkat cells after 48 h of transfection with the control or miR-132-3p mimics as in (**D**). A representative blot of one experiment out of five is shown; protein bands were cropped from the same gel. **(F)** Protein relative levels of p85α, p55α and p50α as in (**E**) after normalization to p150 (n = 5) T-test **P < 0.05; *P < 0.01.

Next, we used a combination of prediction programs to analyze the predicted miRNA binding sites on the 3′UTR of *pik3r1* (Supplementary Table S1). We identified ten canonical binding sites and two unusual sites for miRNAs upregulated during T cell activation in the 3′UTR of *pik3r1*. We focused on the interaction of miR-132-3p with *pik3r1* because the prediction algorithms projected two different binding sites for miR-132-3p in the 3′UTR of the gene; also, because the role of this miRNA in the context of T cell activation had not been previously reported. We generated luciferase reporter vectors of the two regions of the 3′UTR of *pik3r1* containing the two predicted target sites of miR-132-3p. For Site 1, we used the 71-362 fragment; Site 2 contained the 2957-3162 fragment (Fig. 3A). We co-transfected the reporter plasmids into HEK cells either with a control plasmid or a plasmid driving the overexpression of miR-132-3p co-expressing GFP. GFP-positive cells were sorted by flow cytometry and overexpression of miRNA-132 was monitored by RT-qPCR (Fig. 3B). Luciferase signal analysis revealed that both predicted sites are targeted by miR-132-3p, since luciferase levels were lower in cells co-transfected with miR-132-3p plasmid compared to the control plasmid (Fig. 3C). To further assess the functional relationship between miR-132-3p and *pik3r1* gene in T cells, we next transfected miR-132-3p mimics into Jurkat T cells and evaluated the levels of *pik3r1* gene products, the proteins p85α, p55α and p50α that are expressed in this cell line. After 48 h post transfection, miR-132-3p was overexpressed in these cells, significantly reducing p55α and p50α protein levels (Fig. 3D–F). The overexpression by transfection of miR132-3p mimic resulted in levels of the miRNA greater than the endogenous levels of activated T cells. However, the reduction of the protein levels was not so dramatic. Indeed, p85α protein levels were lower in miR-132-3p overexpressing cells although the decrease was milder compared to the other isoforms probably due to the higher stability of this specific isoform. Thus, our data experimentally demonstrate that miR-132-3p inhibits the expression of the products of the *pik3r1* gene in T lymphocytes.

The prediction algorithms that we used projected that the different pik3r1 transcripts would be targeted by 11 of the 12 miRNAs upregulated after T cell activation. Moreover, we detected a downregulation of both its protein products and mRNA transcripts at different time points after initial T cell activation. The specific loss of p85α (with the other regulatory subunits unaltered like p50α) has been previously shown to inhibit the activation of Akt under conditions promoting the differentiation of Th1, Th2 and Th17 cells, but only Th17 differentiation was affected^21^. A recent study described that patients with mutations on PIK3R1 undergo excessive lymphoproliferation and exhibit hyperactive PI3K signaling as a result of the abnormally low level of expression of the mutant p85α^22^. We hypothesize that the decreased expression of *pik3r1* after T cell activation might be regulated by a combination of miRNAs that control that PI3K signaling is precisely dosed during T cell activation. In this context, miR-132-3p would have a cooperative role in T cell activation. It is worth mentioning that signaling downstream pik3r1 was not significantly changed by the overexpression of miR-132-3p (data not shown) further supporting the idea of miR-132-3p cooperative role with other miRNAs. However, our experimental data only provide information about this specific miRNA. In summary, we propose that the modulation of the observed group of miRNAs during T cell activation, including miR-132-3p, finely tunes the availability of gene products to promote the proper activation of T cells.

## Methods

### Mice

C57BL/6 and OT-II mice were bred under specific pathogen-free conditions according to European Commission recommendations at Centro Nacional de Investigaciones Cardiovasculares (CNIC) animal facility. All experimental methods and protocols were approved by CNIC and Comunidad Autónoma de Madrid and followed European Commission guidelines and regulations.

### Cell lines culture and transfection

HEK-293T were cultured in DMEM Medium (SIGMA) supplemented with 10% Fetal Bovine Serum (FBS) (Invitrogen). Cells were co-transfected with Psicheck2 reporter plasmids for *pik3r1* 3′UTR fragments and control GFP plasmid or a miR-132-3p-GFP plasmid (ABM) with Lipofectamine-2000 (Invitrogen) according to manufacturer’s instructions. GFP+ cells were sorted at FACSAria flow cytometer (BD Biosciences) before downstream analysis. Jurkat cells were cultured in RPMI (Sigma) containing 10% FBS (Invitrogen). Jurkat cells were transfected with either miRIDIAN miRNA Mimic negative Control-Dy547 or miRIDIAN miRNA Mimic mmu-miR-132-3p (Dharmacon) by electroporation. Cells were resuspended in Opti-MEM (GIBCO) with 1 μM of mimic and electroporated with Gene Pulser Xcell (Bio-Rad) at 1200 μFa, 240 mV during 30 ms.

### Primary cells isolation, culture and activation

Mouse primary cells were cultured in RPMI 1640 medium supplemented with 10% fetal bovine serum, 50 μM 2-mercaptoethanol and 1 mM sodium pyruvate.

Mouse naive CD4+ T cells were isolated from cell suspensions of lymph nodes or spleen that were incubated with biotinylated antibodies (BD Biosciences) against CD8, CD19, CD25, CD11b, CD11c, CD45R, MHC-II (I-Ab), DX5, IgM, Gr-1 and F4/80, subsequently with streptavidin microbeads and negatively selected in auto-MACS Pro Separator (Miltenyi Biotec) according to the manufacturer’s instructions. Wild type bone marrow cell suspensions were either cultured to obtain conventional DCs or both pDCs and cDCs as described^23, 24^. When indicated, OT-II CD4 T cells were cocultured with the corresponding subset of DCs (8:1T cell/DC ratio) in the presence or absence of chicken ovalbumin (OVA) 323–339 peptide. Polyclonal activation of CD4 T cells was performed with 10 μg ml^−1^ of anti-CD3 plate bound and 2 μg ml^−1^ of anti-CD28 (BD Biosciences).

### Flow cytometry analysis and sorting

Cell samples were analyzed with a BD FACS Canto or BD LSR Fortessa flow cytometers and FACSDiva software (BD Biosciences) and FlowJo software. For mouse CD4 T cells phenotyping to check purity and activation the following antibodies were used against: CD4, CD69, CD62L, CD25, and CD8 coupled with the appropriate fluorophore (BD Biosciences). Cells were sorted on a FACSAria flow cytometer (BD Biosciences). CD4+ T cells from the coculture with DCs were discriminated by staining of MHC-II and CD4 plus CD69.

### Cloning

The two fragments of the 3′UTR of *pik3r1* (sequence from 71 to 362 bp and from 2957 to 3162 (Fig. 3A) were cloned into psiCHECK2 vector (Promega). Fragments were amplified from genomic DNA using Q5 High-Fidelity DNA Polymerase (New England BioLabs) and the corresponding primers (Supplementary Table S1). Each amplified DNA fragment was ligated to the psiCHECK2 vector after digestion with PmeI restriction enzyme (NEB) using the *Gibson Assembly* Master Mix^25^ (NEB) following manufacturer’s instructions.

### Immunoblotting

Total cell extracts were prepared in RIPA lysis buffer and analyzed by Western blotting. The following antibodies were used: anti-alpha Tubulin (DM1A, Sigma), anti-p150^glued^ (BD Transduction Laboratories), rabbit anti-p85a (Millipore), anti ezrin/moesin (ERMs) (90/3) (provided by Heinz Furthmayr, Stanford University, CA). Full immunoblots are provided in Supplementary Figure S4.

### Luciferase UTR reporter assays

HEK cells were lysed after 24 h postransfection and the ratio of Renilla and Firefly luciferase activities was measured by the dual luciferase assay (Promega). Psicheck2 dual luciferase reporter vector comprises the gene of Firefly luciferase as a normalizing gene and the luciferease *Renilla reniformi*s gene downstream of the cloning site.

### RNA isolation

Total RNA was extracted with the miRNeasy mini kit (Qiagen). Purity and concentration were measured in a Nanodrop-1000 spectrophotometer (Thermo Scientific) and RNA integrity using the Agilent 2100 Bioanalyzer.

### microRNA microarrays and analysis

Agilent Mouse miRNA V2 (4 × 44 K) microarray was performed on RNA preparations. miRNA data were normalized based on the VSN-invariant method^26, 27^ using the GeneView files extracted from the Agilent Feature Extraction suite. This method preserves the biological characteristics of the data while stabilizing the variance across all the intensity range based on a fit to some invariant miRNAs (113 in this case). After normalization, only those probes present in at least two samples and with average expression over the 20th percentile of all average expressions were considered for further analysis (198 miRNAs). We used linear models^28^ as implemented in the limma Bioconductor package.

The microarray data have been deposited in NCBI’s Gene Expression Omnibus and are accessible through GEO Series accession number GSE85363.

### Bioinformatic analysis of miRNA target and miRNA seed sequence on 3′UTRs

The targets of the murine microRNAs upregulated after T cell activation were predicted using the Weighted Scoring by Precision (WSP) method^17^. Briefly, the method searches nine databases of predicted interactions for the putative targets of the object miRNAs and finds which of these interactions are among four databases of experimentally validated interactions. Thus, the reliability and precision of each interaction in each database is calculated. An integrated score is calculated for each interaction as the sum of each individual database score multiplied by the precision of that interaction in the specific database.

The sequences of the 3′-UTR of murine pik3r1 gene and those of mature miRNAs upregulated after T cell activation were retrieved from the Ensembl database (release 72, June 2013) and the miRBase database (release 19, August 2012), respectively. Predictions were made using the algorithms miRanda^29^, PITA^30^, FindTar v3.11.12 ^31^, RNAHybrid^32^, and TargetScan v6 ^33^ using parameter default values. The sequences of six, seven and eight nucleotides of each miRNA 5′ end were considered as seeds, starting from the first, second or third nucleotide. Mismatches were not allowed in the canonical seeds but noncanonical seed-matches were also searched.

### RT-qPCR of mature miRNA and messenger RNA

cDNA was synthesized and mature miRNAs were quantified by miRCURY LNA Universal RT microRNA PCR (Exiqon), using miRNA LNA primers (Exiqon) and SYBRgreen PCR master mix (Applied Biosystems). cDNA for mRNA quantification was synthesized using the High Capacity cDNA Reverse Transcription Kit (Applied Biosystems) and quantitative PCR was performed with SYBRgreen PCR master mix (Applied Biosystems) and corresponding primers (Supplementary Table S2). Quantitative miRNA or mRNA expression data were acquired on ABI Prism 7900HT SDS (Applied Biosystems) and further analyzed using BiogazelleQBasePlus software (Biogazelle). Results are expressed in arbitrary units (A.U.) relative to endogenous controls, RNU1A1 and RNU5G RNAs for miRNAs and B-actin and Yhwaz for mRNAs.

## Electronic supplementary material

Supplementary Information
