# Supplementary material for: miRNA profiling during antigen-dependent T cell activation: A role for miR-132-3p
Source: Sci Rep. 2017 Jun 14;7:3508. doi: 10.1038/s41598-017-03689-7 (PMC5471249; doi:10.1038/s41598-017-03689-7)
Supplement: Supplementary file 1 — Supplementary Information [file 41598_2017_3689_MOESM1_ESM.pdf]

SUPPLEMENTARY INFORMATION

miRNA profiling during antigen-dependent T cell activation: A role for miR-132-3p

Cristina Gutiérrez-Vázquez[1,2], Ana Rodríguez-Galán[1,2], Marcos Fernandez-Alfara[2], María Mittelbrunn[2], Fátima Sanchez-Cabo[2], Dannys Jorge Martínez-Herrera[3], Marta Ramírez-Huesca[2], Alberto Pascual-Montano[3] and Francisco Sánchez-Madrid[1,2] *

SUPPLEMENTARY FIGURES:

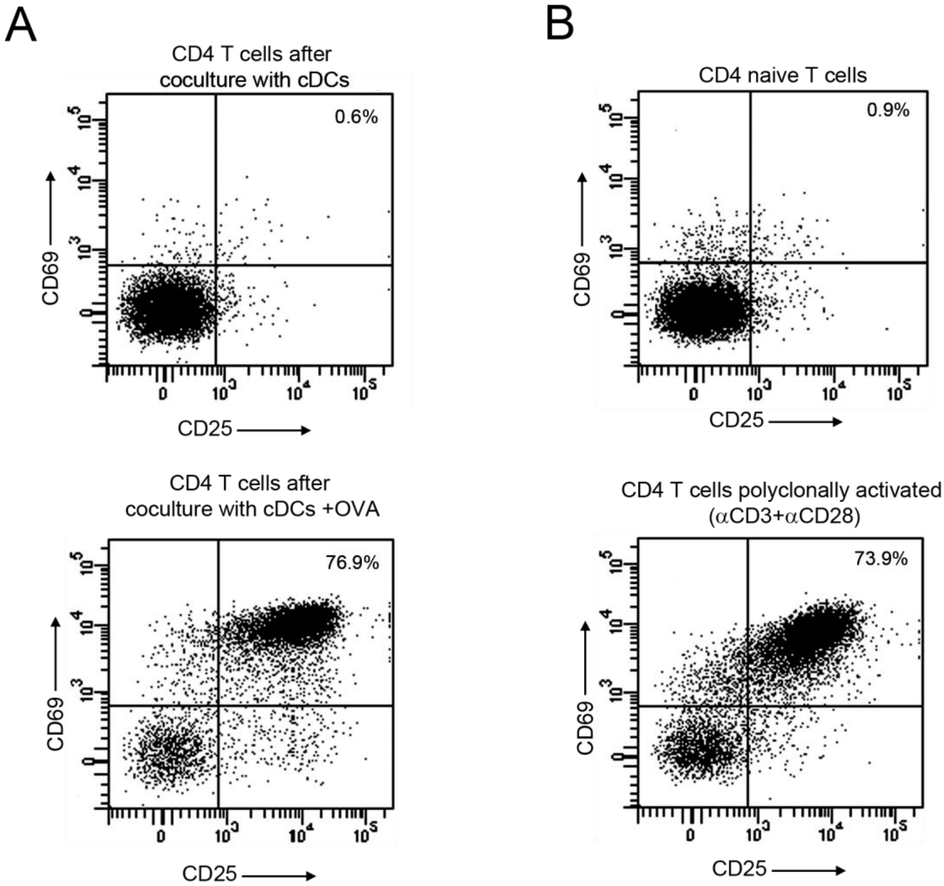

Supplementary Figure S1: CD4 T cell activation after coculture with different DCs subsets. (A) CD4 T cells from OT-II mice were coculture with cDCs in the absence (upper panel) or presence (lower panel) of OVA peptide. CD4 T cells were gated and analyzed for the activation markers CD25 and CD69. **(B)** The same analysis was performed on naïve CD4 T cells (upper panel) or CD4 T cells after polyclonal stimulation with anti CD3 and anti CD28 antibodies (lower panel).

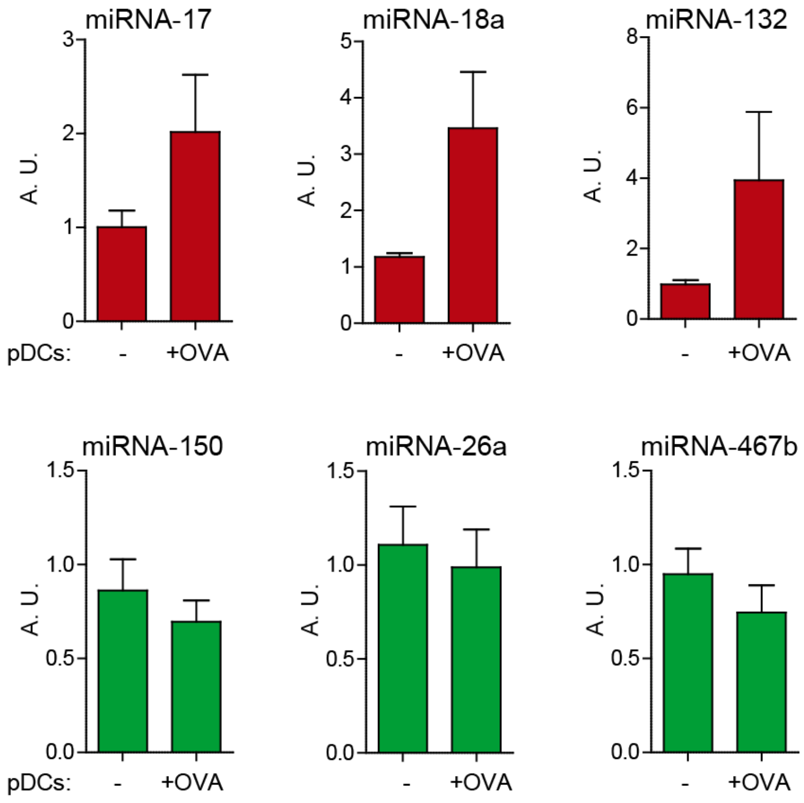

Supplementary Figure S2: miRNA expression after cognate interaction with plasmacytoid DCs. Selected miRNAs detected by microarrays were validated by RT-qPCR. Selected miR-17, miR-18, miR-132, miR-26a, miR-150 and miR-467b miRNAs were analyzed in T cells after their stimulation by plasmacytoid DCs loaded or not with OVA peptide. (n=8).

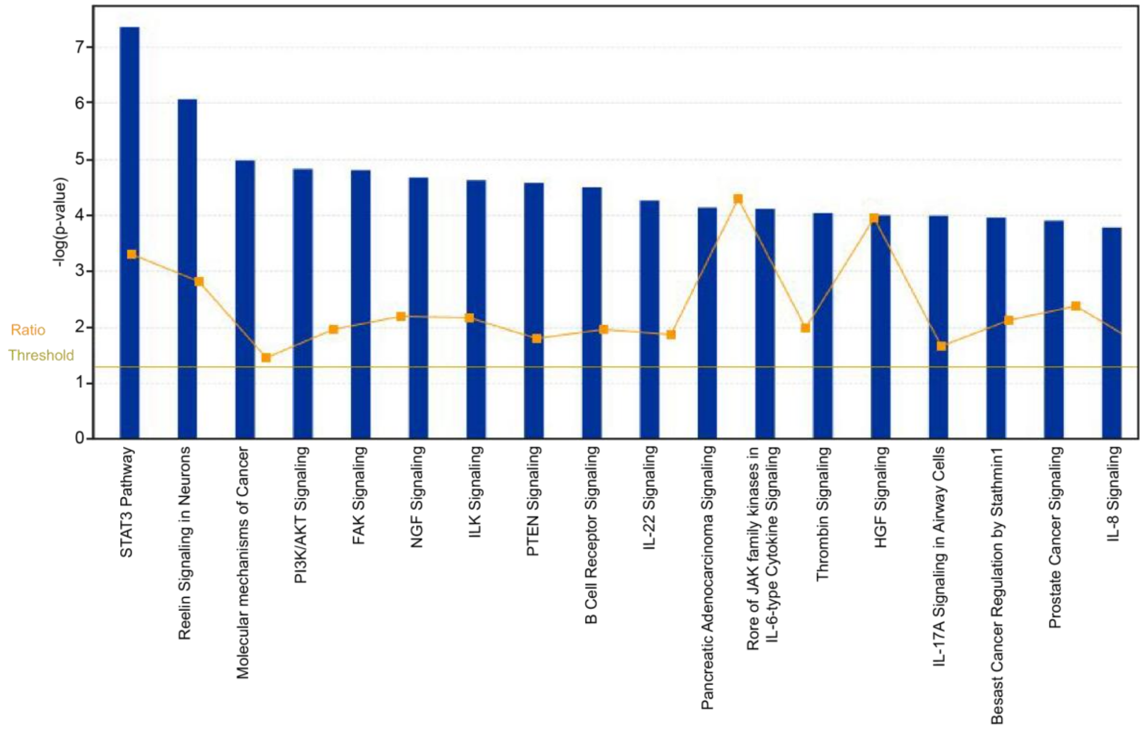

Supplementary Figure S3: Signaling Pathways of upregulated miRNAs Targets. Genes regulated by 7 or more upregulated miRNAs during T cell activation were analyzed with Ingenuity Pathway Analysis to find common pathways of these targets. Those pathways with higher score are shown. Ratio between the number of molecules in our list within the total number of molecules(genes) in that pathway of expression is depicted (orange line).

Related to Figure 2C

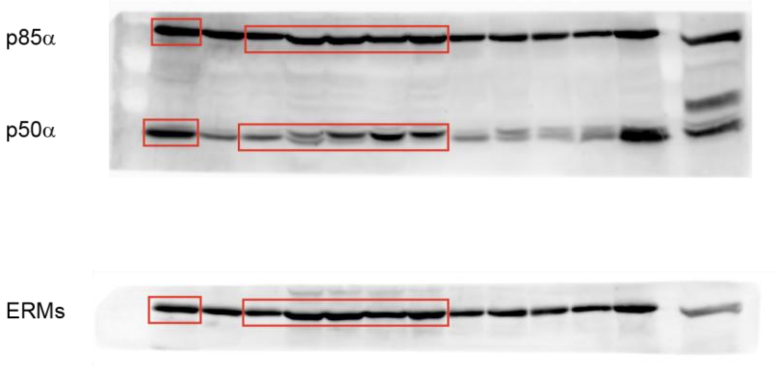

Related to Figure 3E

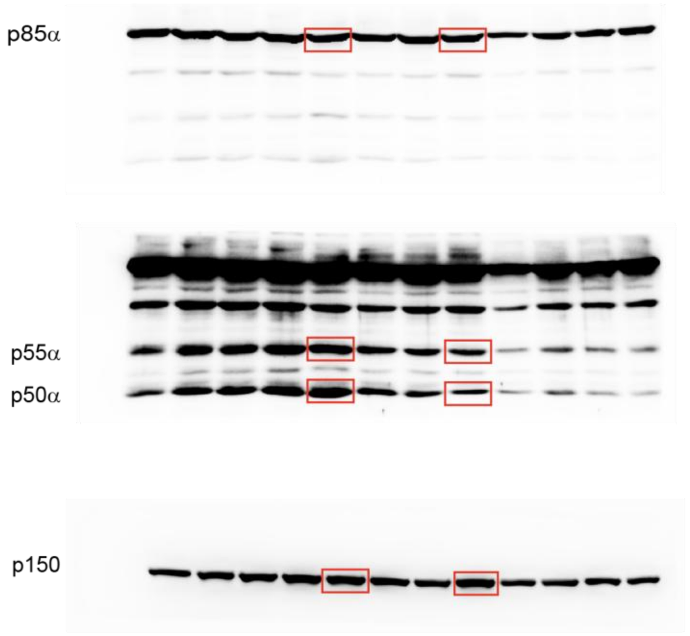

Supplementary Figure S4: Full Immunoblots. Full length immunoblots with indicated areas of selection.

SUPPLEMENTARY TABLES:

Supplementary Table S1

Canonical sites							
**miRNA**	**Start**	**End**	**Seedmatch Sequence**	**Seedmatch type**	**3' pairing**	**# sites**	**Prediction Programs**
mmu-miR-106a-5p	1377	1383	GCACTTT	7mer-m8	-	1	targetscan, miranda, findtar
**mmu-miR-132-3p**	**3082**	**3088**	**GACTGTT**	**7mer-m8**	**-**	**2**	targetscan, miranda, findtar
**mmu-miR-132-3p**	**175**	**181**	**GACTGTT**	**7mer-m8**	**-**		targetscan, miranda, findtar
mmu-miR-146a-5p	3359	3365	GTTCTCA	7mer-m8	13-17	1	targetscan, rnahybrid, findtar
mmu-miR-17-5p	1377	1383	GCACTTT	7mer-m8	-	1	targetscan, miranda, findtar
mmu-miR-20a-5p	1377	1383	GCACTTT	7mer-m8	-	1	targetscan, miranda, findtar
mmu-miR-21a-5p	2400	2406	TAAGCTA	7mer-m8	-	3	targetscan, findtar
mmu-miR-21a-5p	904	910	ATAAGCT	7mer-m8	-		targetscan, miranda, findtar
mmu-miR-21a-5p	2937	2943	TAAGCTA	7mer-m8	-		targetscan, findtar
mmu-miR-34a-5p	2299	2305	ACTGCCA	7mer-m8	-	1	targetscan, findtar
Unusual sites							
**miRNA**	**Start**	**End**	**Seedmatch Sequence**				
mmu-miR-21a-5p	1154	1162	TGATAAGCT				
mmu-miR-155-5p	1442	1465	AGTTTGGTAGTCATTA GCAATTAA				

Supplementary Table S1: pik3r1 3'UTR miRNA binding sites sequences. The predicted binding sites for upregulated miRNAs at 3'UTR of pik3r1 mRNA were analyzed with a prediction tool that combines different prediction programs available at the time of the analysis. Seedmach type, sequence and location as well as the specific programs that predict this binding are shown for each interaction.

Supplementary Table S2:

**qPCR primers**	
**Oligo Name**	**Sequence (5' to 3')**
Mouse YWHAZ (Forward)	CGTTGTAGGAGCCCGTAGGTCAT
Mouse YWHAZ (Reverse)	TCTGGTTGCGAAGCATTGGG
Mouse b Actin (Forward)	CAGAAGGAGATTACTGCTCTGGCT
Mouse b Actin (Reverse)	TACTCCTGCTTGCTGATCCACATC
Mouse B2M (Forward)	TTCTGGTGCTTGTCTCACTGA
Mouse B2M (Reverse)	CAGTATGTTCGGCTTCCCATTC
Mouse Pik3r1 v1 (Forward)	ACACCACGGTTTGGACTATGG
Mouse Pik3r1 v1 (Reverse)	GGCTACAGTAGTGGGCTTGG
Mouse Pik3r1 v2 (Forward)	ATTTCACCCCCTACTCCCAAG
Mouse Pik3r1 v2 (Reverse)	AGTCGAACATTCCAGTCCTTT
**Cloning primers**	
**Primer**	**Sequence (5' to 3')**
Site 1 (primer A)	*GCCCGGGAATTCGTTT*CCAGCCCGACCTGTGAAC
Site 1 (primer B)	*GGCCGCTCTAGGTTT*GGCCTCTTTGTCCCTGCA
Site 2 (primer A)	*GCCCGGGAATTCGTTT*GGAGGGTTGGGACCTTGTGTT
Site 2 (primer B)	*GGCCGCTCTAGGTTT*G ACCTGTACTGGACATCTGCTTG

Supplementary Table S2: Primers used in this study
